# The changing epidemiology of SARS-CoV-2

**DOI:** 10.1126/science.abm4915

**Published:** 2022-03-10

**Authors:** Katia Koelle, Michael A. Martin, Rustom Antia, Ben Lopman, Natalie E. Dean

**Affiliations:** 1 Department of Biology, O. Wayne Rollins Research Center, Emory University, Atlanta, GA 30322, USA.; 2 Graduate Program in Population Biology, Ecology, and Evolution, Emory University, Atlanta, GA 30322, USA.; 3 Department of Epidemiology, Rollins School of Public Health, Emory University, Atlanta, GA 30322, USA.; 4 Gangarosa Department of Environmental Health, Rollins School of Public Health, Emory University, Atlanta, GA 30322, USA.; 5 Department of Biostatistics and Bioinformatics, Rollins School of Public Health, Emory University, Atlanta, GA 30322, USA.

## Abstract

We have come a long way since the start of the COVID-19 pandemic—from hoarding toilet paper and wiping down groceries to sending our children back to school and vaccinating billions. Over this period, the global community of epidemiologists and evolutionary biologists has also come a long way in understanding the complex and changing dynamics of severe acute respiratory syndrome coronavirus 2 (SARS-CoV-2), the virus that causes COVID-19. In this Review, we retrace our steps through the questions that this community faced as the pandemic unfolded. We focus on the key roles that mathematical modeling and quantitative analyses of empirical data have played in allowing us to address these questions and ultimately to better understand and control the pandemic.

By February 2020, it was clear that a global coronavirus pandemic had begun to unfold. The rapid rise in COVID-19 cases before lockdown measures went into effect in Wuhan, China, on 23 January 2020 showed how efficient human-to-human transmission of severe acute respiratory syndrome coronavirus 2 (SARS-CoV-2) was. By January 2020, the increasing number of documented cases of SARS-CoV-2 outside of China further indicated that regional containment of this virus was going to be extremely unlikely. At this time, epidemiologists and infectious disease modelers began to characterize the key features of the 2019 novel coronavirus (2019-nCoV)—subsequently renamed SARS-CoV-2 in February 2020—that were critical for assessing its containment potential and for projecting its spread at various geographic scales (Fig. 1).

The ability to contain an emerging pathogen depends in part on its basic reproduction number *R*_0_ (1), defined as the average number of secondary cases arising from a primary case in a completely susceptible population (2). No active containment efforts are required when *R*_0_ < 1, but higher values of *R*_0_ make containment more difficult (2). Estimating *R*_0_ for an emerging disease is not straightforward; doing so robustly requires calculating how quickly the virus is spreading through a population and having a measure of the time between one infection and the next, which is known as the generation interval (Fig. 2). Early epidemiological analyses therefore focused on estimating the rate of viral spread from reported Wuhan case data. The generation interval was parameterized using data from previous coronavirus outbreaks [SARS-CoV-1 and Middle East respiratory syndrome coronavirus (MERS-CoV)] and from the small number of transmission pairs that had been identified in early SARS-CoV-2 outbreaks. This led to estimates of *R*_0_ ranging between 2 and 4 before the implementation of lockdown measures in Wuhan. The differences between these estimates were largely the result of differences in how the generation interval was parameterized (3), which underscores the importance as well as the scarcity of high-resolution outbreak datasets to accurately infer the generation interval and, thereby, the value of *R*_0_.

Beyond *R*_0_, the ability to contain an emerging pathogen depends on the capacity to isolate infectious individuals (1). This in turn depends on the proportion of infections that are asymptomatic or only mildly symptomatic (and therefore easily missed during surveillance) and their transmission potential. It also depends on the potential for presymptomatic transmission from individuals who are infectious before developing symptoms. Early on, it was unclear how common asymptomatic and mildly symptomatic infections were—i.e., how large the bottom of the infection “iceberg” might be. Epidemiological models were interfaced with reported infection data from China to infer the proportion of SARS-CoV-2 cases that went undocumented before 23 January 2020 (yielding an estimate of 86%) (4). In February and March of 2020, mass screening and surveillance of passengers aboard cruise ships and flights where outbreaks occurred yielded some of the first estimates of the proportion of infections that remained asymptomatic: 18 to 31% (5, 6). It was also apparent from early data that mild disease, most notably in younger age groups, was a common outcome. Crucially, contact tracing studies further revealed high infectiousness around the time of symptom onset, with frequent transmission from presymptomatic and mildly symptomatic individuals (7–9), and transmission also occurring (although less commonly) from fully asymptomatic individuals (10). These findings were sobering in terms of their implications for containing the pandemic. Unlike SARS-CoV-1 and MERS-CoV, SARS-CoV-2 was discerned from the start to have the potential to cause a global pandemic with tremendous public health impacts.

## Flattening the curve

SARS-CoV-2 had spread to many regions of China and across the world by late February 2020. Although there were a few countries, such as New Zealand and Vietnam, that achieved almost complete control of the initial spread through rigorous surveillance efforts (11, 12), community transmission quickly took off in many other countries. Mathematical scenario models predicted the devastation that would result if no control measures were implemented (13, 14). Coupled with accounts coming out of northern Italy, London, New York City, and the other earliest-affected regions, projections from these models quickly led to government-imposed restrictions to curb viral spread (15). Nonpharmaceutical interventions (NPIs) included lockdowns, social distancing measures, and, eventually, the use of face masks. Adoption of NPIs during the first wave of the pandemic served to flatten the curve—that is, to extend the period over which cases occurred. Flattening the curve was desirable for three reasons. First, it would prevent the health care system from being overwhelmed because the peak number of beds occupied at any one time would be lower under a flatter curve. Second, it would slow the momentum of the outbreak, reducing the overshoot of cases after the outbreak peak (16). Third, it allowed time to improve clinical care strategies and capacity and to evaluate therapeutics.

With some NPI measures in place, modeling efforts turned toward forecasting the future dynamics of COVID-19. Some of the most public-facing forecasting that was reported in April and May of 2020 were the controversial projections made by the Institute for Health Metrics and Evaluation (IHME). These projections relied on statistical modeling approaches, which can provide accurate short-term projections but are likely to fail for longer-term predictions because they do not incorporate the process of transmission (17). Indeed, IHME projections at this time predicted little viral circulation by midyear 2020. Mechanistic epidemiological modeling studies instead predicted a slowdown of viral spread while tough NPI measures were in place but a resurgence of cases after NPI relaxation (14).

As the first wave provided epidemiologists with longitudinal SARS-CoV-2 case data, COVID-19 death data, and virus sequence data, the effect that NPIs had on reducing viral spread started to be quantitatively assessed. These analyses found that NPIs were effective at flattening the curve and even at temporarily reducing reproduction numbers to the extent that circulation levels declined (18, 19). These findings mirrored those from studies of historical pandemics, such as the 1918 H1N1 influenza pandemic, which found that NPIs were successful at reducing disease transmission (20). Counter-factual models were used to quantify how many cases and deaths could have been averted by different policies, such as earlier social distancing measures (21).

Although NPIs were effective at reducing viral spread at the population level, infection risk was not reduced equally across populations. Health care workers and frontline workers (e.g., first responders) were at particularly high infection risk in April and May of 2020 owing to their close contact with SARS-CoV-2–positive individuals. Many other essential occupations were also found to carry increased risk, including public-facing jobs, such as taxi and bus drivers, and jobs in poorly ventilated and crowded settings, such as factories and meatpacking plants. Crowded living settings, where social distancing was not possible, also carried increased risks for infection (22). Even with NPIs in place, large outbreaks occurred in communal-living facilities, such as nursing homes, homeless shelters, jails, and prisons (23). Risks were exacerbated by poor sanitation and inadequate health care. Sharp differences in case rates also became clearly visible across socioeconomic and racial and ethnic groups (24).

As anticipated, the widespread use of NPIs, where implemented, exacted considerable social and economic costs. To facilitate the reopening of the economy, researchers sought to distinguish highly effective NPIs from those that could potentially be discarded. Key to assessing the effectiveness of different NPIs was understanding the routes of viral transmission. How easily did the virus spread between individuals through contaminated surfaces (fomites) compared with direct transmission by aerosols or droplets? Although an early study from April 2020 found that SARS-CoV-2 could remain viable on surfaces, such as plastic, for days (25), subsequent studies indicated that transmission occurred primarily by direct transmission from infected individuals rather than through fomites (26). Contact tracing studies also revealed the higher risk of direct transmission occurring in closed and crowded places and close-contact settings (27), known by Japanese public health officials as the three Cs. Large outbreaks were traced to night-clubs, karaoke bars, and choir practices—all indoor settings characterized by loud vocalization. Statistical analyses found that limitations on public gatherings and workplace closures were positively associated with lower levels of SARS-CoV-2 spread (28, 29), corroborating the role of direct transmission in closed areas in facilitating the transmission of SARS-CoV-2.

Large outbreaks detected by contact tracing, coupled with emerging virus sequence data, quickly led to the recognition of substantial transmission heterogeneity between individuals. Studies using data from the first wave found that ~10% of cases were responsible for 80% of secondary infections (19, 30), a relatively high level of heterogeneity when compared with other human pathogens (31). Explanations for superspreading dynamics ranged from variable contact patterns across individuals (32); high-risk settings conducive to spread (23); and biological factors, such as high viral load (32–34). Notably, if superspreading individuals or events could be systematically identified, then control efforts could plausibly focus on mitigating spread in a more targeted manner. An influential review of the efficacy of face masks also indicated that mask wearing had an appreciable effect on reducing transmissibility per contact by reducing transmission of infected respiratory particles (35). Mask wearing, particularly by infected individuals with high viral loads (i.e., source control), therefore had the potential to reduce viral spread among the population.

Epidemiological modeling also pointed toward ways in which surveillance and control efforts could be improved upon to allow for relaxation of broadly applied NPIs. For example, modeling studies showed that traditional contact tracing and digital contact tracing had the potential to slow viral spread in the presence of relaxed lockdown measures (36, 37). Another study found that increasing testing frequency and reporting speed for SARS-CoV-2 would be more effective at limiting the spread of the virus than exclusively relying on highly sensitive tests with slower reporting timelines (38, 39). Several modeling studies highlighted the trade-offs involved for different timelines of NPI relaxation and how uncertainty in NPI effects and implementation errors may thwart our ability to optimally control disease spread through the use of NPIs (40, 41).

## Riding out the waves

The initial wave of infections was followed by subsequent waves, reminiscent of those seen in the years after the 1918 influenza pandemic (42). Factors that contribute to waves of transmission include evolutionary changes in the virus as well as changes in host immunity and behavior.

Theory would predict that soon after the introduction of a zoonotic pathogen, virus adaptation would result in increased transmission between humans. The rapid spread of the D614G variant [with a glycine residue (G) at position 614 in the spike protein replacing an aspartic acid residue (D)] between March and May of 2020 pointed toward the possibility of virus adaptation occurring (43) (Fig. 3A). That this replacement reflected virus adaptation was supported by experiments showing that the variant had enhanced replication in human bronchial and nasal airway epithelial cell cultures as well as increased replication and transmissibility in in vivo infections of hamsters and ferrets (44). Quantitative analyses of population-level SARS-CoV-2 sequence data from the UK also detected a transmission advantage of D614G in humans (45).

As time progresses during a pandemic, immunity from natural infection builds up in a population. For SARS-CoV-2, the initial hope was that population immunity in areas with large first waves would greatly reduce future transmission. The level of herd immunity required for protecting a population is roughly 1 − 1/*R*_0_ (46). Based on an *R*_0_ of 3, this yielded an estimate of 67% for SARS-CoV-2, but transmission heterogeneity was theorized to significantly lower the herd immunity threshold (47). However, as early as August 2020, documented accounts of reinfection indicated that immunity to SARS-CoV-2 may only transiently protect from infection (Fig. 3B) (48). Studies of the endemic human coronaviruses (HKU1, OC43, 229E, and NL63) at this time also found evidence for repeated reinfections (49) and for antigenic evolution (50, 51) (Fig. 3C), which put a damper on hopes that herd immunity would rapidly bring an end to the pandemic.

Throughout the second half of 2020, as we were gaining a better understanding of the virus’s transmission characteristics and the duration of immunity, SARS-CoV-2 cases ebbed and flowed, with waves peaking at different times in different countries and even in different regions in the same country. The causes of these temporal changes in incidence were likely the result of a combination of factors, including changes in NPIs, behavioral changes, seasonal changes (influenced by weather and holiday schedules), and the development of transmission-reducing immunity through natural infection followed by its waning. Quantifying the interactions between these factors and their relative roles remains an important area for study, critical for projecting the longer-term dynamics of SARS-CoV-2 (52, 53).

## Vaccines and variants

By December 2020, vaccines against SARS-CoV-2 were developed, trialed, and approved for emergency use—a timeline unparalleled in the history of vaccinology. Epidemiological factors facilitated the accelerated timeline of these pivotal trials. With little acquired immunity to SARS-CoV-2 and a high incidence of COVID-19 in mid-2020 when trials were underway, the studies reached their prespecified end points rapidly and ahead of anticipated schedule. As is widely appreciated now, the trials demonstrated excellent protection against COVID-19 and severe outcomes, like hospitalization and death. But questions remained even after the vaccines were authorized for use (Fig. 4): How long will protection last? Will the vaccines protect against new virus variants? Will they prevent transmission? Although randomized trials continued to accrue data for a period of several months after their completion, observational studies, including cohorts and case-control designs, were critical for monitoring real-world vaccine effectiveness along these different dimensions.

Many decisions about the vaccine rollout, including policy-relevant questions such as which groups to target initially, had to be made before such knowledge was available, so epidemiological modeling served a key role in exploring the potential impact of decisions under different assumptions. One initial question was whether to maximize coverage by offering a single dose of a two-dose series to as many people as possible or to maintain fidelity to the trial protocols of two doses (spaced 3 to 4 weeks apart for the mRNA vaccines). Modeling studies, parameterized with trial data that showed apparent protection starting 10 to 14 days after receipt of a first dose, indicated that in the short term, a one-dose strategy is likely preferable for limiting disease at the level of the population (54). However, in the longer term, if vaccine efficacy is substantially lower with one dose than with two, a two-dose strategy might be preferable, depending on how rapidly the virus was infecting people and how quickly the vaccine could be rolled out. Concerns about the potential for SARS-CoV-2 to evolve immune escape in the context of incomplete immunity from a single dose was also addressed through quantitative modeling and reasoning (54, 55). On the basis of early trial data and modeling results, the UK promoted a strategy whereby second doses were delayed compared with the trial interval. In doing so, high coverage was achieved rapidly among >50 year olds, and a decoupling became apparent in which high case counts did not result in parallel hospitalization or mortality trends in postvaccination pandemic waves. This observation, predicted by scenario modeling studies (56), showed the value of vaccines in preventing severe disease. Indeed, postintroduction evaluations indicated that vaccines offered ~90% protection against hospitalization and death (57, 58).

Vaccine-related questions broadened to ones regarding the extent to which vaccination could reduce transmission (Fig. 4). Vaccines may avert transmission by two mechanisms—by preventing infection and by rendering breakthrough cases less infectious. Landmark observational studies from mid- to late 2021 demonstrated protection against infection, although to a lesser degree than against disease or severe outcomes (58, 59). Some studies also indicated that vaccinated individuals experiencing breakthrough infections may have lower viral loads or a more rapid viral decline and thus perhaps less potential for onward transmission (60–62) [but see (63, 64)]. The most holistic understanding of vaccine performance against transmission comes from household studies that offer insight into infection-blocking and transmission-blocking mechanisms. One such study estimated vaccine efficacy against infectiousness of breakthrough cases to be 23% (65). When combined with protection against infection, the reduction in transmission was estimated to be 92%.

Complicating the assessment of SARS-CoV-2 vaccine efficacy was the evolution of new variants of concern around the time that vaccines were rolled out. In late 2020, the B.1.1.7 lineage [now known as Alpha in the World Health Organization’s (WHO’s) variant naming scheme] was detected and rapidly spread in the UK. Several of the mutations harbored by this lineage significantly altered the viral phenotype, including enhanced binding to angiotensin-converting enzyme 2 (ACE2) (66), with consequences for transmissibility (67). Quantification of Alpha’s selective advantage based on viral sequence data indicated that it had 1.5 to 2 times the reproduction number of other lineages circulating at that time (68).

Soon after the emergence of the Alpha lineage, a different viral lineage, B.1.617.2 (which, along with its descendant lineages, make up the Delta variant of concern), was fueling a large wave of SARS-CoV-2 infections in India (69). This lineage rapidly swept through India and became dominant worldwide. In vitro analyses indicated that convalescent human serum bound less efficiently to Delta than to ancestral lineages and that Delta had higher replication efficiency (70). Consistent with these findings, a modeling study found that Delta’s growth advantage at the population level stemmed from a combination of Delta’s higher transmissibility and its reduced sensitivity to host immune responses generated through prior infection (69). Fortunately, postintroduction vaccine effectiveness studies found that protection against symptomatic disease was largely maintained against both the Alpha and Delta variants (58, 71, 72). Subsequent studies clearly established that vaccine effectiveness against infection and symptomatic disease wane over time (73) such that additional vaccine doses (boosters) can be beneficial, particularly to older-aged individuals (74).

**Fig. 1.**
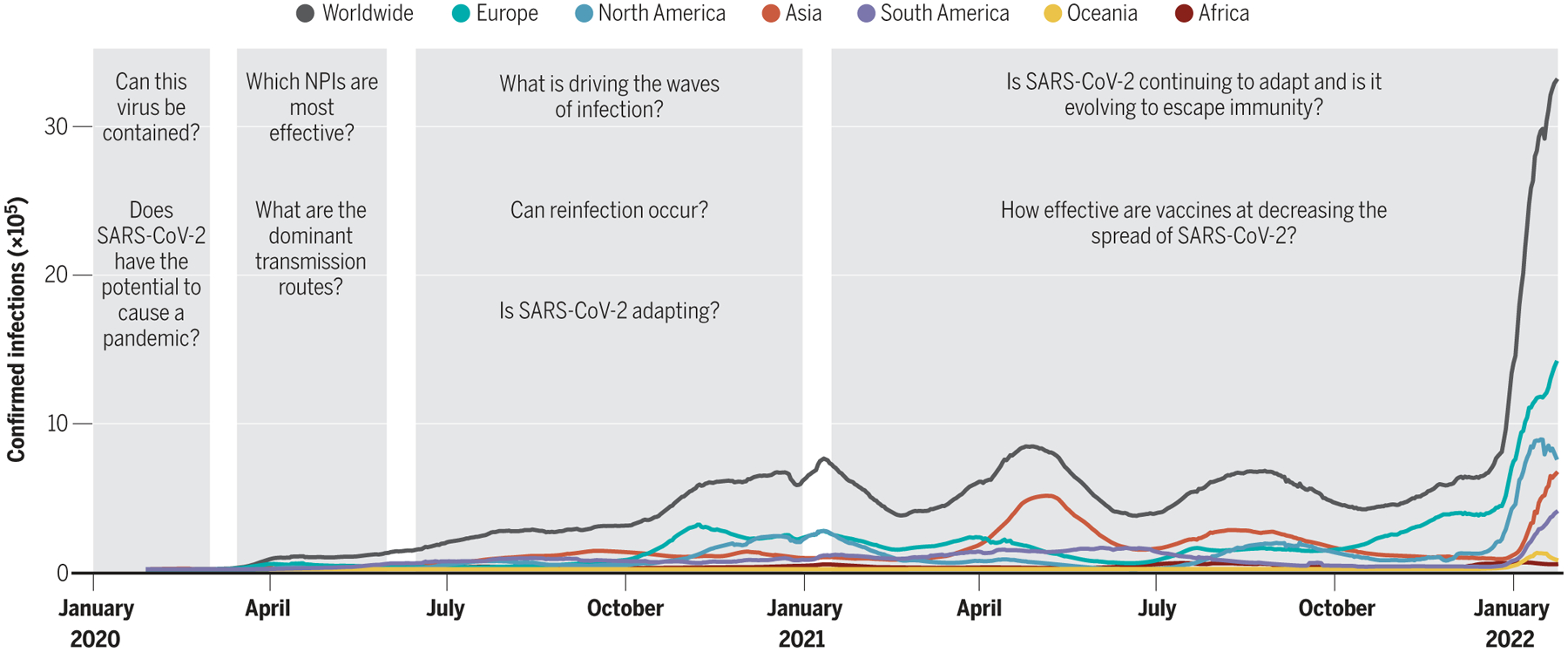
Timeline of changing epidemiological questions asked as the SARS-CoV-2 pandemic unfolded. Confirmed infections are shown by continent as well as on a worldwide scale. Gray bars indicate the time periods discussed in this Review (the emerging pandemic, flattening the curve, riding out the waves, and vaccines and variants).

**Fig. 3.**
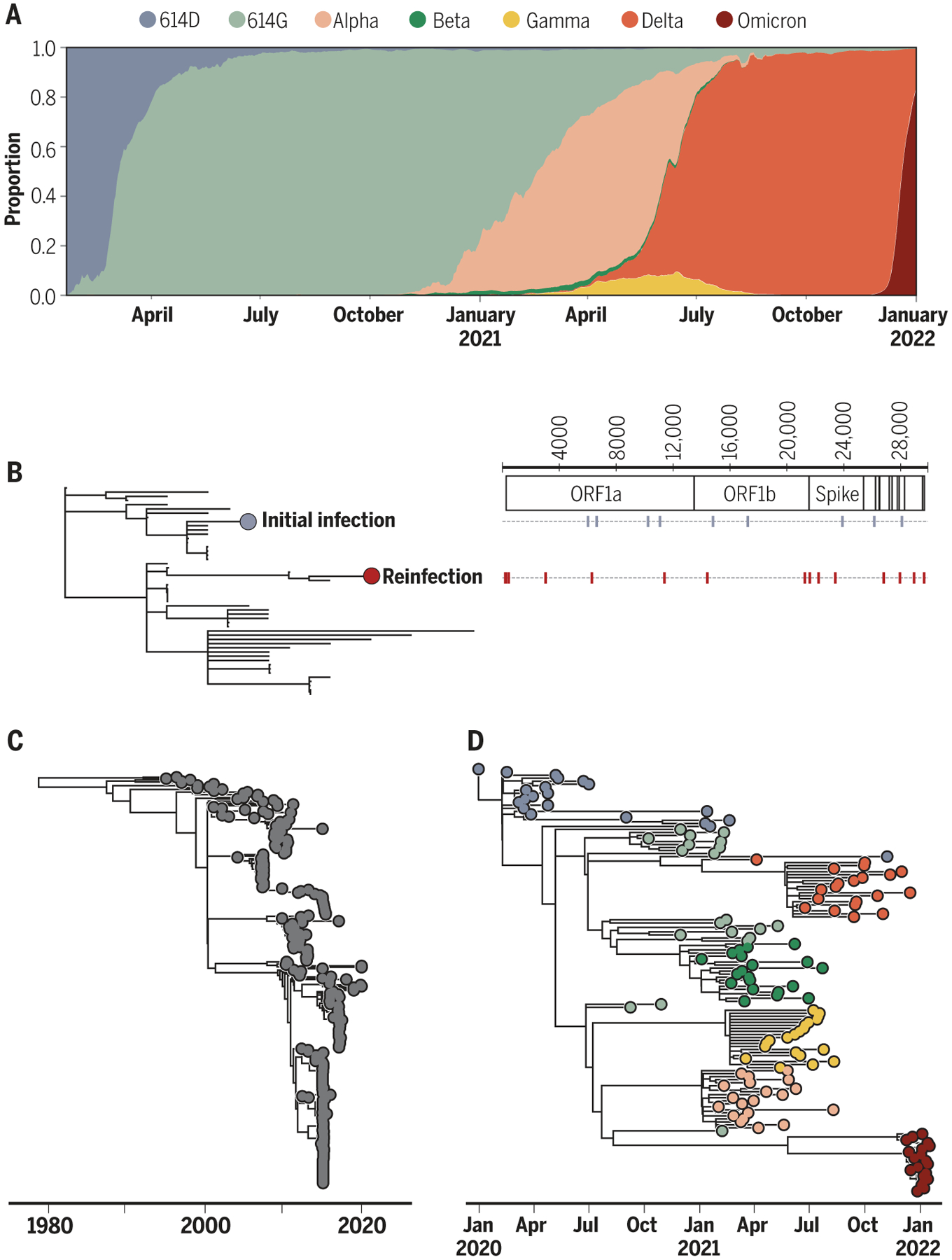
Coronavirus sequence data have informed epidemiological understanding of SARS-CoV-2. (**A**) The frequencies of SARS-CoV-2 variants of concern over time. The first indication that SAR-COV-2 was adapting to humans was the population-level replacement of the 614D allele with the 614G allele in early 2020. Variant frequencies on the *y* axis are calculated on the basis of SARS-CoV-2 sequence data deposited in GISAID (Global Initiative for Sharing Avian Influenza Data). Only major, globally circulating variant lineages are shown. (**B**) An example of viral sequencing that has provided evidence of reinfection with SARS-CoV-2. Reproduced with permission from (48). Samples from a patient in the context of circulating viral genetic variation indicate that the observed secondary infection is not a reactivation of a latent infection but is instead a reinfection. Schematic on the right shows the substitutions present in the primary and secondary infection viral samples. ORF, open reading frame. (**C**) A phylogeny of seasonal human coronavirus OC43 (lineage A). Phylogenetic analysis points toward antigenic evolution in this viral population. Reproduced with permission from (50). (**D**) A phylogeny inferred from SARS-CoV-2 sequence data showing the evolutionary relationships between the variant lineages included in (A).

**Fig. 4.**
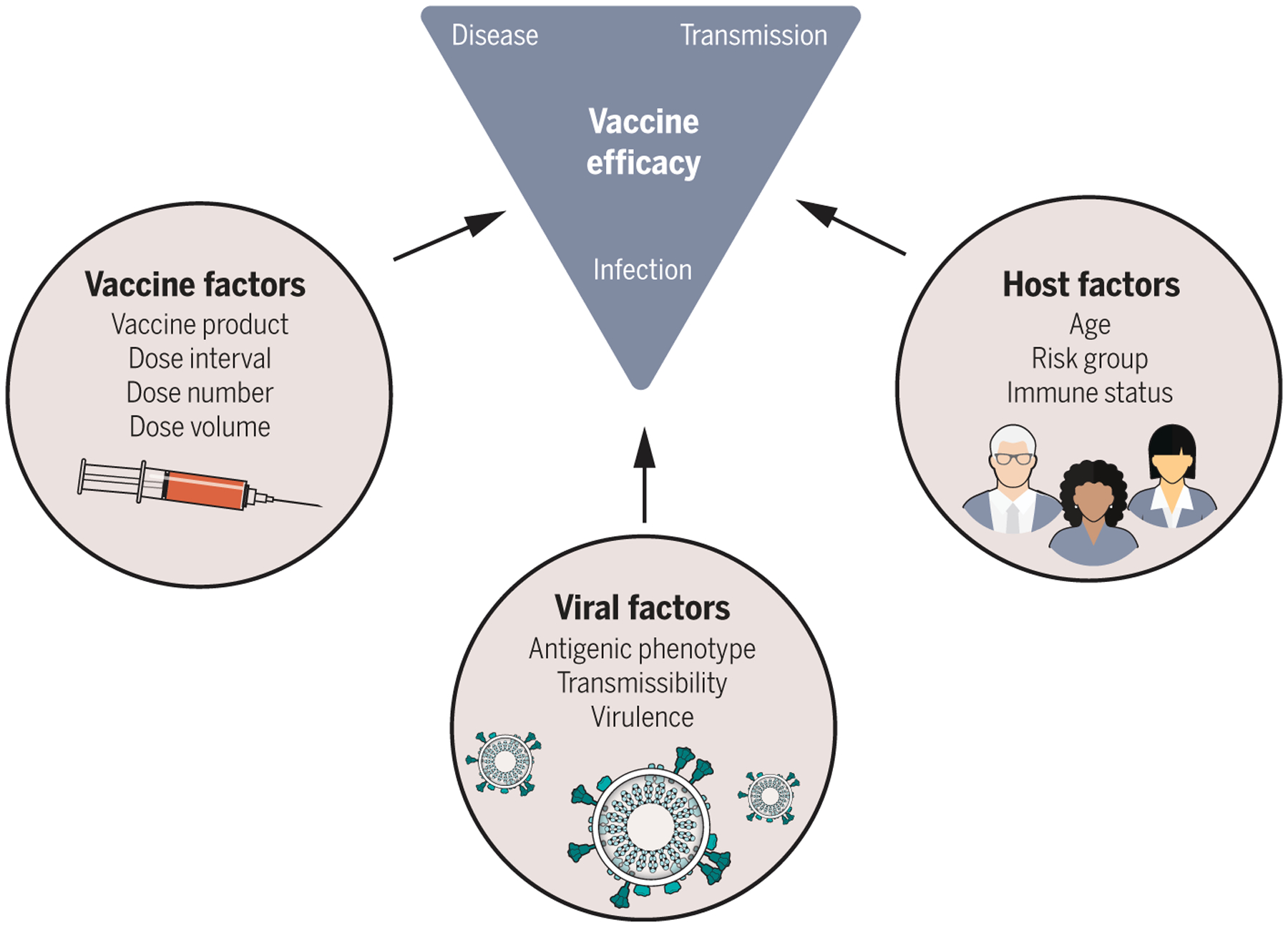
Vaccine, host, and viral factors that affect vaccine efficacy. Each of the factors shown were investigated as potential moderators of vaccine efficacy in clinical trials and postintroduction observational studies. Scenario modeling projected the population-level effects of vaccination by incorporating data-driven assumptions on how vaccination affects susceptibility to infection, disease, and severe outcomes like hospitalization and death and how these effects differed by host factors, such as age. A key determinant of population impact of vaccination is the effect on transmission, which is a combination of infection-blocking and transmission-reducing effects of vaccines. As data emerged, mainly from observational studies, assumptions about the infectiousness of vaccine breakthrough cases were incorporated into models. With the evolution of Delta and then Omicron, variant-specific vaccine efficacy and the potential for viral immune escape became critical quantities to understand. With this array of vaccine, host, and viral data on the effects of vaccination on vaccine recipients, epidemiological models could project both the direct and indirect effects of vaccination on the population-level spread of SARS-CoV-2.

In November 2021, researchers in South Africa noticed a rising number of COVID-19 cases with S gene target failure (SGTF), indicative of an expanding viral subpopulation harboring the Δ69–70 spike gene deletion. Shortly thereafter, circulation of a new variant was confirmed through whole-genome sequencing. Based on its mutational profile and its extremely rapid spread in South Africa, the WHO designated this variant as one of concern, naming it Omicron (B.1.1.529). In December 2021, this variant spread rapidly throughout the world, resulting in more confirmed infections than ever before (Fig. 1) and rapidly replacing the Delta variant (Fig. 3A). Omicron’s mutational profile relative to the Wuhan-Hu-1 reference genotype consists of more than 30 mutations in the spike glycoprotein (75), many of which are predicted to enable immune escape. Early studies confirmed the ability of Omicron to evade antibodies from previous infection and vaccination (76) and indicated that it can use an alternative cell entry pathway, which improves its ability to infect cells in the upper respiratory tract (77). Animal models further indicated that infection with Omicron may be less clinically severe than infection with previous variants (78). Experimental predictions have been borne out in epidemiological studies, in which Omicron has been shown to be better at reinfecting previously infected people (79) and less clinically severe compared with Delta (80). Nevertheless, the rapid expansion of Omicron has resulted in the largest spike in confirmed SARS-CoV-2 infections thus far in many countries, and spikes in deaths attributed to the virus have followed. Vaccine effectiveness against severe disease and death, however, has remained high with the new variant, with Omicron-caused deaths concentrated among unvaccinated individuals.

The emergence of Omicron, and Alpha before it, raises important evolutionary questions regarding the sources of new, genetically divergent variants. At their time of emergence, both variants harbored more mutations than would have been expected given the rate at which SARS-CoV-2 evolves in the human population. Omicron’s distant evolutionary relationship to other SARS-CoV-2 variants is notable (Fig. 3D). One possibility for the evolutionary origin of these variants is in an alternative host: SARS-CoV-2 has been observed in a number of domestic and wild animal species (as a result of reverse zoonotic events), and these new variants may have evolved in one of these alternative hosts and spilled back into the human population. Alternatively, these variants may have evolved in immunocompromised or immunosuppressed individuals with chronic SARS-CoV-2 infections, given documented examples of the extent to which adaptive evolution can happen in these types of infection (81). Both putative sources of new variants remain unobserved in human population surveillance sequencing data, which highlights the need for more comprehensive monitoring strategies to identify variants of concern earlier and to identify and stop potential variants of concern before they are widespread.

## Looking ahead

SARS-CoV-2 has been difficult to control since the start of the pandemic, and the recent spread of Omicron has only amplified this challenge. It is inevitable that SARS-CoV-2 will become an endemic pathogen in the human population. However, its impact on our health and daily lives will change considerably over time (82). Governments and public health officials must transition to a long-term strategy of dealing with SARS-CoV-2. Vaccines and therapeutics, including outpatient treatment to prevent progression of disease, are highly valuable tools. Yet the challenge ahead remains formidable, with transmission levels high even in areas with widespread vaccination and prior waves of infection, and many open questions remain about the future epidemiological impact of this virus.

The most evolutionarily successful variants of SARS-CoV-2 so far have evolved greater transmissibility relative to more ancestral variants. Although higher transmissibility will result in more infections, increasing population-level immunity from infections and vaccinations might make these infections less severe in terms of health impact. However, the evolutionary pressures facing the virus, and thus its patterns of adaptive evolution, will change. Variants, such as Omicron, that gain much of their transmission advantage from evading immunity may become the norm, as is the case with seasonal influenza.

The evolution of SARS-CoV-2 virulence in terms of how harmful or deadly it is has been a topic of debate since the beginning of the pandemic (83). Evolutionary theory has pointed out that we should not expect evolution toward lower virulence (84), and the last two variants of concern have demonstrated that there is not a clear, consistent trend in SARS-CoV-2 virulence evolution: Although Delta is thought to be slightly more virulent than previous variants, Omicron is less so. Although the virulence of SARS-CoV-2 may still evolve over time (in a direction that is not easily predicted), we expect the infection fatality ratio to decline for other reasons, including rising population immunity. As noted above, the available SARS-CoV-2 mRNA vaccines confer protection against severe disease, even where breakthrough infections occur. Vaccine boosters will further help in protecting against severe disease. Monoclonal antibodies and antivirals to be taken shortly after symptom onset will similarly reduce the risk of severe disease and death. Over time, population immunity will result in a younger age at first infection, which will reduce average infection severity and likely lead to milder infections later in life. Parallels can be drawn to the seasonal corona-viruses, where individuals are usually infected very early on in life and where secondary infections are milder than primary infections because of preexisting immunity (85).

## Stepping back

The most pressing scientific questions have changed over the past 2 years, reflecting the complexity of this pandemic (Fig. 1). Early questions focused on placing the novel pathogen on a map with respect to its pathogenicity and transmissibility. Modeling assumptions relied heavily upon experiences with SARS-CoV-1 and MERS-CoV and on a limited number of SARS-CoV-2 datasets. Over time, we replaced broad assumptions with improved understanding. The rapid spread of the virus and the explosive waves of infection that ensued necessitated swift reactions, and questions shifted from short-term to longer-term considerations. We made a major leap forward with the deployment of safe and effective vaccines, but new variants and waning immunity dampened our hopes for greater control of transmission. Moving forward, key questions remain about the propensity of this virus to further adapt to our human population and the health impact of endemic transmission. Although our work as scientists studying this pathogen remains far from over, we recognize all that has been achieved to support the unifying mission of a safer and healthier world.

## Figures and Tables

**Fig. 2.**
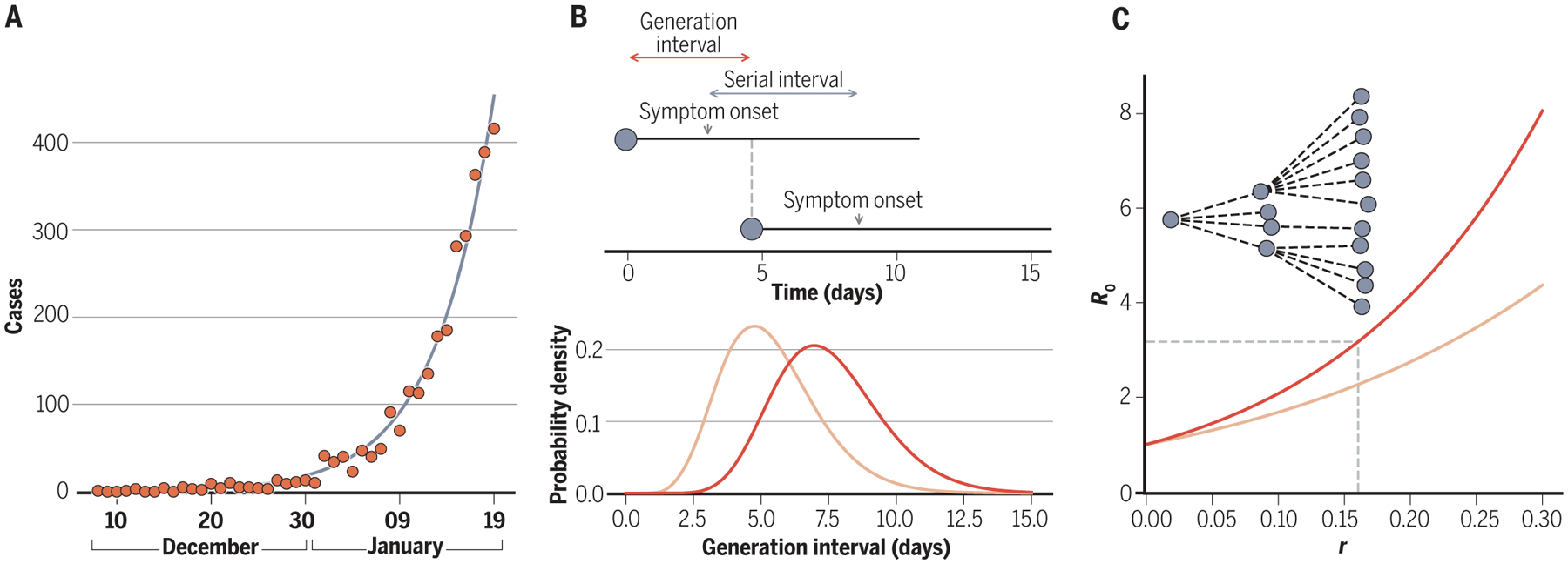
Estimation of the basic reproduction number *R*_0_ from case data and detailed outbreak data. (**A**) Exponential growth models are fit to observed case data to estimate the speed at which a virus spreads through a population. For SARS-CoV-2 spread in Wuhan, early estimates of the exponential growth rate *r* fell in the range of 0.10 to 0.20 per day (3), yielding epidemic doubling times of ~3.5 to 7 days. (**B**) Outbreak data are used to estimate the viral serial interval, defined as the time between symptom onset of an index case and symptom onset of the index case’s contacts. This serial interval is often used as an approximation for the viral generation interval. For SARS-CoV-2, an early Wuhan estimate of the mean serial interval was 7.5 days (86) (distribution in red). An example of a generation interval distribution with a smaller mean is shown in light orange. (**C**) The basic reproduction number *R*_0_ can be calculated from the exponential growth rate *r* and the distribution of the generation interval using an equation derived from demographic analyses (87). Red and light orange curves, corresponding to the distributions in (B), show how the *R*_0_ estimates depend on the generation interval. The transmission chain at the top illustrates an outbreak with an *R*_0_ = 3 pathogen.
